# Hemodynamic effects and tolerance of dobutamine for myocardial dysfunction during septic shock: An observational multicenter prospective echocardiographic study

**DOI:** 10.3389/fcvm.2022.951016

**Published:** 2022-09-09

**Authors:** Keyvan Razazi, Vincent Labbé, Laurent Laine, Alexandre Bedet, Guillaume Carteaux, Nicolas de Prost, Florence Boissier, Francois Bagate, Armand Mekontso Dessap

**Affiliations:** ^1^AP-HP, Hôpitaux Universitaires Henri-Mondor, Service de Médecine Intensive Réanimation, Créteil, France; ^2^INSERM, Institut Mondor de Recherche Biomedicale (IMRB), Univ Paris Est Créteil, Créteil, France; ^3^Faculté de Médecine de Créteil, Institut Mondor de Recherche Biomedicale (IMRB), GRC CARMAS, Université Paris Est Créteil, Créteil, France; ^4^Département Médico-Universitaire APPROCHES, AP-HP, Hôpital Tenon, Service de Médecine Intensive Réanimation, Sorbonne Université, Paris, France; ^5^Hôpital Delafontaine, Service de Réanimation, Saint-Denis, France; ^6^CHU de Poitiers, Service de Médecine Intensive Réanimation, Poitiers, France

**Keywords:** septic shock, myocardial depression, dobutamine, mortality, echocardiography

## Abstract

**Background:**

The role of dobutamine during septic shock resuscitation is still controversial.

**Methods:**

The aim of this prospective multicentre study was to comprehensively characterize the hemodynamic response of septic shock patients with systolic myocardial dysfunction to incremental doses of dobutamine (0, 5, 10, and 15 μg/kg/min).

**Results:**

Thirty two patients were included in three centers. Dobutamine significantly increased contractility indices of both ventricles [crude and afterload-adjusted left ventricular (LV) ejection fraction, global LV longitudinal peak systolic strain, tissue Doppler peak systolic wave at mitral and tricuspid lateral annulus, and tricuspid annular plane excursion) as well as global function indices (stroke volume and cardiac index) and diastolic function (increased e' and decreased E/e' ratio at lateral mitral annulus). Dobutamine also induced a significant decrease in arterial pressure and cardiac afterload indices (effective arterial elastance, systemic vascular resistance and diastolic shock index). Oxygen transport, oxygen consumption and carbon dioxide production all increased with dobutamine, without change in the respiratory quotient or lactate. Dobutamine was discontinued for poor tolerance in a majority of patients (*n* = 21, 66%) at any dose and half of patients (*n* = 15, 47%) at low-dose (5 μg/kg/min). Poor tolerance to low-dose dobutamine was more frequent in case of acidosis, was associated with lower vasopressor-free days and survival at day-14.

**Conclusion:**

In patients with septic myocardial dysfunction, dobutamine induced an overall improvement of echocardiographic parameters of diastolic and systolic function, but was poorly tolerated in nearly two thirds of patients, with worsening vasoplegia. Patients with severe acidosis seemed to have a worse response to dobutamine.

## Introduction

Circulatory failure is one of the hallmark alterations in septic shock and involves a variable combination of hypovolemia, vasoplegia and myocardial dysfunction. Septic myocardial dysfunction was first described by Parker et al. in 1984 (1). In recent studies using echocardiography, systolic dysfunction was observed in one third (when assessed by left ventricle ejection fraction, LVEF) and more than two-thirds (when assessed by speckle tracking-derived LV longitudinal peak systolic strain) of patients during septic shock (2). Diastolic dysfunction is also common and is a strong independent predictor of early mortality in septic shock (3).

The surviving sepsis campaign recommends the use of dobutamine in patients who show evidence of persistent hypoperfusion despite adequate fluid loading and the use of vasopressor agents (4). However, the role of dobutamine during septic shock resuscitation is still controversial since most clinical studies have been performed in an unselected population including patients with increased or decreased systolic function (5, 6). Dobutamine failed to improve sublingual microcirculatory and hepatosplanchnic peripheral perfusion parameters or lactate levels in a randomized placebo crossover study (7). In addition, dobutamine may worsen cardiac diastolic function (via its tachycardic effect) and hypotension (via its vasoplegic effect) during septic shock. We hypothesized that despite its beneficial effects on systolic function, dobutamine may alter diastolic function and worsen hypotension in patients with septic myocardial dysfunction.

The aim of this study was to comprehensively characterize the response of patients with septic myocardial dysfunction to incremental doses of dobutamine in terms of macrocirculation, cardiac function (including loading conditions, systolic and diastolic function), microcirculation (mottling), and tissue hypoxia (indirect calorimetry and lactatemia).

## Materials and methods

### Patients

Patients who met septic shock criteria (as defined according to the Sepsis-3 definition) (8), either from community-acquired or nosocomial infections, were prospectively screened in three intensive care units (ICU) of Greater Paris in France. Norepinephrine was the first-choice vasopressor therapy (used to target a mean arterial pressure of 65 mmHg or more). Inclusion criteria were the presence of septic myocardial dysfunction [as defined by depressed LVEF (<45%) at echocardiography on the first or second day of septic shock onset] with ongoing signs of hypoperfusion despite adequate mean arterial pressure and correction of hypovolemia with absence of fluid responsiveness (see study procedure), mandating the introduction of dobutamine as per the physician decision, and in agreement with surviving sepsis campaign recommendations (9). Non-inclusion criteria were chronic heart failure (defined as a baseline LVEF below 45%), severe valvulopathy, moribund state, tachycardia with heart rate >130 bpm, patients already receiving an inotropic agent, hemodynamic instability with mean arterial pressure <65 mmHg despite norepinephrine infusion, and unavailability of trained operators or echocardiography system.

Patient's severity was evaluated by the Mac Cabe and Jackson score for underlying diseases (10), the SAPS II (Simplified Acute Physiologic Score) for acute illness at ICU admission (11), and the SOFA (Sequential Organ Failure Assessment) score for organ dysfunction at septic shock onset (12).

### Study procedure

Tests to predict fluid responsiveness included: i) a “clinical” variable (either pulse pressure or stroke volume variation with a threshold of 12%) (13) and an “echocardiographic” variable (either variation of inferior or superior vena cava diameter) (14); in case of discrepancy between the clinical and echocardiographic variable, the physician performed a fluid challenge: rapid infusion of 250–500 mL saline with fluid unresponsiveness defined as the lack of cardiac output increase (i.e., 10% increase), with fluid challenge, assessed by echocardiography) (15). Dobutamine was started only in patients with fluid unresponsiveness.

An infusion of dobutamine was started at a rate of 5 μg/kg/min, and was sequentially increased to 10 μg/kg/min and 15 μg/kg/min after 30 min intervals, in case of good tolerance. Poor tolerance of dobutamine was defined as one of the following: (i) worsening hypotension (mean arterial pressure <65 mmHg with a decrease of 10 mmHg or more as compared to baseline value or the need to increase norepinephrine infusion by at least 0.5 mg/hour to maintain a mean arterial pressure ≥65 mmHg); ii) severe tachycardia (new-onset atrial fibrillation or sinus tachycardia >130 beats per minutes with an increase of 10 beats per minute or more as compared to baseline value). Hemodynamic measurements were performed before dobutamine start and at the end of each step and included: arterial pressure and heart rate, diastolic shock index = $\frac{heart\;rate\;\left(bpm\right)}{diastolic\;arterial\;pressure\;\left(mmHg\right)}$ (16), mottling score (17), echocardiography, arterial blood gases with lactate, and indirect calorimetry [using Carescape R860 (General Electric Healthcare, USA), to assess oxygen consumption, carbon dioxide production, and energy expenditure] (18). Ventilatory settings, sedative and fluids infusions were kept constant throughout the dobutamine titration protocol, as well as vasopressor dose (unless poor tolerance). The dobutamine titration was discontinued in case of poor tolerance.

### Echocardiography

Serial transthoracic echocardiographies were performed by trained operators (competence in advanced critical care echocardiography) with a standard procedure (19). In case of poor echogenicity, trans-esophageal echocardiography was performed (*n* = 6). All measures were averaged over a minimum of three cardiac cycles (five to ten in case of non-sinus rhythm).

#### Assessment of contractility and loading conditions

The primary outcome was the change in diastolic function, as assessed by tissue Doppler early (*e'*) diastolic wave velocity at the lateral mitral valve annulus. LV filling pressures were also assessed using *E*/*A* and *E*/*e* ratios from pulsed-wave Doppler early (*E*) and late (*A*) and tissue Doppler early (*e'*) wave velocities at the lateral mitral valve annulus (20, 21).

Afterload was assessed using the following indices: i) systolic arterial pressure (which is often used as a surrogate of LV afterload in clinical practice); (22) ii) systemic vascular resistance (the most commonly used measure of vascular tone) (23) $=\frac{80^{*}mean\;arterial\;pressure\;(mm\;Hg)}{cardiac\;output\;(L.\min^{-1})};$ iii) effective arterial elastance (to reflect the pulsatile component of peripheral load)(24) $=\frac{0.9^{*}Systolic\;arterial\;pressure\;\left(mm\;Hg\right)}{stroke\;volume\;\left(mL\right)}$;

LV systolic function was assessed using indices obtained by two-dimensional echocardiography (LVEF), tissue Doppler imaging (tissue Doppler peak systolic wave at the lateral mitral valve annulus), speckle tracking imaging (global longitudinal peak systolic strain of the LV, peak of systolic and early diastolic longitudinal strain rate) (2). An afterload-adjusted LVEF was assessed as recently proposed (25), using a simple nonlinear approach $=\;LVEF^{*}\sqrt{effective\;arterial}\;elastance$. Ventriculoarterial coupling was defined as the ratio of effective arterial elastance to left ventricular end-systolic elastance, which was estimated by using the single beat method of Chen et al. (26, 27).

We measured the velocity time integral in the LV outflow tract and the LVOT diameter, which allowed us to calculate LV stroke volume and cardiac index.

We also assessed i) Stroke work (SW) = 0.9^*^systolic arterial pressure (*mmHg*)^*^stroke volume (*ml*), ii) potential energy (PE)$=\frac{0.9^{*}Systolic\;arterial\;pressure\;{\left(mm\;Hg\right)}^{*}LVend\;systolic\;volume\;\left(ml\right)}{2}$ iii) LV pressure-volume area (PVA) = *SW*+*PE iiii*) Left ventricular work efficiency (which is the ratio of external work to total cardiac work during cardiac cycle) $=\frac{100^{*}SW\;}{PVA}$.

This study was approved by an Institutional Review Board (CPP Ile de France-V), as a component of standard care. Written and oral information about the study was given to the patients or families as per French law.

### Statistical analysis

The data were analyzed using GraphPad Prism (Version 8.4.3) and IBM SPSS Statistics (Version 24). Normal distribution was checked using the Kolmogorov-Smirnov test. The global effect of dobutamine was assessed by the Friedman test (or mixed model analysis in case of missing data) followed by *post-hoc* paired Wilcoxon test with the Benjamini-Hochberg's correction. Continuous data were expressed as medians [25th−75th percentiles] or mean (± standard deviation), as appropriate, and were compared using the non-parametric Kruskal-Wallis test followed by pairwise Mann-Whitney test. Categorical variables, expressed as percentages, were evaluated using the Chi-square test or Fisher exact test. Two-tailed *p*-values < 0.05 were considered significant. Using tissue Doppler early (e′) diastolic wave velocity at the lateral mitral valve annulus as the primary measure of outcome focused on diastolic function, we calculated that a sample size of at least 33 patients would have a 90% power to detect a 20% decline in that variable with dobutamine titration, considering a baseline e' of 8 cm.s^−1^ with a standard deviation of 2 cm.s^−1^ in our previous cohort (2). Twenty recordings (from twenty separate patients) were randomly selected from the study. The same sets of recordings were analyzed separately by two different ultrasonographers to assess inter-analyser reproducibility (28). The reproducibility was expressed as per the British Standards Institution coefficient (twice the standard deviation of the differences in repeated measurements) (29). This coefficient is directly related to the 95% limits of agreement. It is expressed in the measurement units and is the smallest significant difference between repeated measurements.

## Results

### Patient characteristics

From June 2015 to April 2019, among 57 patients screened for myocardial dysfunction during septic shock, 25 were excluded because of one of the following reasons: chronic heart failure (*n* = 1), already receiving an inotropic agent (levosimendan or adrenaline, *n* = 2), moribund state (*n* = 2), unavailability of trained operators or echocardiography system (*n* = 5), heart rate >130 bpm (*n* = 9), mean arterial pressure <65 mm Hg despite norepinephrine infusion (*n* = 1), and final diagnosis of cardiogenic shock without evidence of sepsis (*n* = 5). Thus, the present study comprises 32 patients (19 men and 13 women), with 28, three and one patient included in each center. Clinical characteristics, comorbidities and organ failures are shown in Table 1. Dobutamine titration was performed after a median of 1 [0-1] day of septic shock onset. The doses of 5, 10, and 15 μg/kg/min of dobutamine could be achieved in 32 (100%), 18 (56%), and 11 (34%) patients, respectively.

**Table 1:** Characteristics of patients with septic shock and myocardial dysfunction.

**Clinical characteristics and comorbidities**	**Patients *n* = 32**
Age, years	67 [57–76]
Male gender	19 (59%)
Body mass index, kg/m^2^	23 [19–27]
SAPS II at ICU admission	64 [50–76]
Hypertension	4 (13%)
Cancer or hematological malignancy	7 (22%)
Cirrhosis	1 (3%)
**Organ failures and hemodynamics**	
SOFA score at ICU admission	11 [10–14]
Norepinephrine treatment	32 (100%)
Arterial blood lactate at admission, mmol/L	3.9 [3–6.4]
Infection source Pulmonary	18 (56%)
Nosocomial infection	10 (31%)
Mechanical ventilation	31 (97%)
Tidal Volume, mL/kg predicted body weight	6.0 [5.7–6.2]
Plateau pressure, cm H_2_O	18 [16–22]
Positive end expiratory pressure, cm H_2_O	8 [5–10]
Acute respiratory distress syndrome	25 (78%)
Fluid administration before dobutamine infusion, ml	2750 [1,500–3,750]
Atrial fibrillation before dobutamine infusion	4 (13%)
SOFA score at dobutamine initiation	12 [10–14]
Delay between between admission and dobutamine infusion, hours	34 [7–23]
Delay between shock onset and dobutamine initiation, hours	29 [6–20]
Femoral central venous catheter	21 (66%)
Femoral arterial catheter	16 (50%)
Death in ICU	15 (47%)

Data are number (percentage) or median [1st−3rd quartile].

SAPS, simplified acute physiologic score; ICU, intensive care unit; SOFA, Sequential Organ Failure Assessment. Respiratory variables were collected just before dobutamine infusion.

### Hemodynamics

Tables 2, 3 summarizes the hemodynamic, echocardiographic, calorimetric and arterial blood gas responses to dobutamine. Results of reproducibility of some echocardiographic variables are reported in Supplementary Table S4.

**Table 2:** Hemodynamic and metabolic response during dobutamine titration in patients with shock and septic myocardial dysfunction.

	**Dobutamine dose**			
	**0 μg.kg^−1^.min^−1^ (*n* = 32)**	**5 μg.kg^−1^.min^−1^ (*n* = 32)**	**Maximal dose§ (*n* = 32)**	***P-*value§**
**Macrocirculation**				
Dose of norepinephrine, μg.kg^−1^.min^−1^	1.3 [0.5;2.3]	1.4 [0.6–2.4]	1.4 [0.6–2.4]	0.08
Dose of norepinephrine, mg/h	5.1 [1.6–9.0]	5.1 [2.2–9.0]	5.3 [2.4–9]	0.08
Heart rate, bpm	101 [81–119]	112 [88–122]*	117 [95–126]*	<0.001
Mean arterial pressure, mmHg	73 [69–79]	68 [59–74]*	64 [56–74]*	<0.001
Diastolic arterial pressure, mmHg	58 [54–64]	51 [44–60]*	49 [44–59]*	<0.001
Diastolic shock index, bpm. mmHg^−1^	1.7 [1.4- 2.0]	2.1 [1.7-2.7]*	2.1 [1.7-2.7]*	<0.001
**Afterload**				
Systolic arterial pressure, mmHg	109 [100–120]	103 [90–114]	100 [87–111]*	0.03
Effective arterial elastance mmHg.mL^−1^	2.6 [2.1–3.2]	2.1 [1.6–2.9]*	2.0 [1.5–2.7]*	<0.001
Systemic vascular resistance, mmHg.L^−1^.min	1,584 [1,320–2,125]	1,087 [815–1,473]*	999 [763–1,387]*^†^	<0.001
**Mottling score**	0 [0–2]	0 [0–1]	0 [0–1]	0.05
**Arterial blood gases**				
pH	7.26 [7.19–7.34]	7.29 [7.20–7.34]	7.29 [7.21–7.35]	0.91
PaCO_2_, mmHg	37 [30–44]	37 [30–42]	37 [31–41]	0.99
PaO_2_/FiO_2_ ratio, mmHg	209 [122–324]	209 [119–345]	214 [119–331]	0.56
SaO_2_, %	97 [94–98]	97 [92–98]	97 [94–98]	0.56
Lactates, mmol/L	2.5 [1.5–3.7]	2.2 [1.6–3.5]	2.4 [1.6–3.5]	0.62
**Oxygen metabolism**				
TaO_2_, ml/ min^−1^.m^−2^	310 [270–396]	393 [295–510]*	438 [295–546]*^†^	<0.001
VO_2_, ml/min	243 [173–278]	247 [193–304]	265 [195–323]	0.01
VCO_2_, ml/min	178 [134–188]	176 [137–196]	183 [141–206]	0.04
Respiratory quotient	0.70 [0.66–0.74]	0.70 [0.64–0.75]	0.69 [0.62–0.75]	0.66
Energy expenditure, kcal/day	1,572 [1,252–1,767]	1,547 [1,346–1,896]	1,602 [1,400–1,999]	0.08

Data are median [1st−3rd quartile] or mean (±standard deviation); §The maximal dose of dobutamine was 5, 10, and 15 μg.kg^−1^.min^−1^ in 32, 18, and 11 patients, respectively; §Friedman test or mixed model analysis;

*p < 0.05 as compared to baseline (0 μg.kg^−1^.min^−1^) by *post-hoc* Wilcoxon paired test with Benjamini-Hochberg's correction;^†^p < 0.05 as compared to dobutamine 5 μg.kg^−1^.min^−1^ by *post hoc* Wilcoxon paired test with Benjamini-Hochberg's correction; TaO_2_, oxygen transport, VO_2_ oxygen consumption determined by indirect calorimetry, VCO_2_: carbon dioxide production determined by indirect calorimetry; see text for definitions.

**Table 3:** Echocardiography parameters during dobutamine titration in patients with shock and septic myocardial dysfunction.

	**Dobutamine dose**			
	**0 μg.kg^−1^.min^−1^ (*n* = 32)**	**5 μg.kg^−1^.min^−1^ (*n* = 32)**	**Maximal dose§ (*n* = 32)**	***P-*value§**
Respiratory variation of inferior vena cava, %	5 [0–11]	2 [0–17]	8 [0–20]	0.34
**Diastolic function**				
E/A ratio at mitral valve	0.94 [0.70–1.13]	0.84 [0.69–1.09]	0.84 [0.71–1.12]	0.15
E-wave deceleration time, ms	162 [115–233]	151 [136–215]	152 [128–340]	0.96
e' at lateral mitral annulus	6.9 (±3.7)	8.3 (±4.3)*	8.3 (±4.5)*	0.004
E/e' ratio at lateral mitral annulus	11.2 (±8.3)	9.8 (±6.9)*	9.9 (±6.6)	0.008
Peak of early diastolic longitudinal strain rate	0.67 [0.57–0.78]	0.87 [0.78–1.13]*	0.93 [0.78–1.29]*	0.005
**Contractility**				
Global LV longitudinal peak systolic strain, %	−8.4 [−10.6 to −7.2]	−10.2 [−14.2 to −8.0]	−10.4 [−14.6 to −8.7]*	0.01
Peak of systolic longitudinal strain rate	−0.72 [−0.94 to −0.57]	−1.1 [−1.2 to −0.82]*	−1.2 [−1.4 to−0.88]*	0.002
s' at mitral lateral annulus, cm.s^−1^	8.0 [5.9–10.7]	9.0 [7.0–12.5]	10.0 [7.8–12.9]*	0.002
LVEF, %	30 [25–40]	40 [30–50]*	45 [35–60]*	<0.001
Adjusted LVEF, %	41 [33–59]	47 [37–63]*	53 [42–67]*	0.006
LV end systolic elastance, mmHg.mL^−1^	1.5 [1.0–1.8]	1.3 [1.0–1.8]	1.3 [1.1–2.0]	0.56
**RV function**				
Tricuspid annular plane excursion, mm	15 [13–17]	17 [12–19]	17 [14–22]*	0.02
s' at tricuspid lateral annulus, cm/s	11.0 [8.0–12.0]	12.8 [11.1–15.7]*	15.0 [11.1–17.0]*	<0.001
RV dilatation (RV/LV area ratio)	0.6 [0.5–0.6]	0.6 [0.5–0.6]	0.6 [0.5–0.6]	0.35
**Global function**				
Stroke volume index, mL.m^−2^	22 [17–27]	27 [19–30]*	28 [19–30]*	<0.001
Cardiac index, L.min^−1^.m^−2^	2.1 [1.7–2.7]	2.9 [2.0–3.6]*	3.1 [2.1–3.6]*^†^	<0.001
Ventricular–arterial coupling	1.8 [1.4–2.3]	1.7 [1.1–2.4]	1.5 [1.1–2.4]	0.82
Stroke work (mmHg mL)	3,500 [2,915–4,908]	3,905 [3022–5,250]	4,010 [2,954–4,961]	0.93
Potential energy (mmHg mL)	905 [463–1,486]	1,239 [641–3408]*	2,638 [1,255–5,749]*^†^	0.02
LV pressure-volume area (mmHg mL)	4,528 [3,257–6,076]	5,143 [3,975–8,566]*	7,059 [4,450–11,103]*^†^	<0.001
LV efficiency (%)	82 [75-86]	75 [67-82]*	73 [50-73]*^†^	<0.001

Data are median [1st−3rd quartile] or mean (±standard deviation); §The maximal dose of dobutamine was 5, 10, and 15 μg.kg^−1^.min^−1^ in 32, 18, and 11 patients, respectively; §Friedman test or mixed model analysis; *p < 0.05 as compared to baseline (0 μg.kg^−1^.min^−1^) by *post-hoc* Wilcoxon paired test with Benjamini-Hochberg's correction;^†^p < 0.05 as compared to dobutamine 5 μg.kg^−1^.min^−1^ by *post hoc* Wilcoxon paired test with Benjamini-Hochberg's correction; LV left ventricle; LVEF, left ventricle ejection fraction; RV, right ventricle; E, blood Doppler early diastolic wave; A, blood Doppler late diastolic wave; e', tissue Doppler early diastolic wave; s', tissue Doppler peak systolic wave.

#### Macrocirculation and cardiac function

Dobutamine induced a decrease in mean arterial pressure and an increase in heart rate. All contractility indices of both ventricles were increased with dobutamine (including crude LVEF, afterload-adjusted LVEF, global LV longitudinal peak systolic strain, longitudinal systolic strain rate, tissue Doppler peak systolic wave at mitral and tricuspid lateral annulus, and tricuspid annular plane excursion), while all afterload parameters were decreased (including systolic arterial pressure, effective arterial elastance, systemic vascular resistance). Dobutamine also improved diastolic function (with increased longitudinal diastolic strain rate, e' and decreased E/e' ratio at lateral mitral annulus) and global cardiac function (with increased cardiac index), but with non-significant change in ventricular–arterial coupling and decrease in LV efficiency.

#### Mottling and tissue hypoxia

There was a trend toward decreased mottling score with dobutamine titration, but few patients had significant mottling. Arterial blood gases and lactate levels did not change during dobutamine titration, whereas oxygen transport, oxygen consumption and carbon dioxide production both increased, with a stable respiratory quotient. Energy expenditure increased with the maximal dose of dobutamine.

### Clinical tolerance

During dobutamine titration, 21 (66%) patients had a poor tolerance leading to discontinuation in 15 patients at 5 μg/kg/min, four patients at 10 μg/kg/min, and two patients at 15 μg/kg/min. The reasons for discontinuation were worsening hypotension in the majority of patients (*n* = 18, including 14 at 5 μg/kg/min, two at 10 μg/kg/min and two at 15 μg/kg/min), severe sinus tachycardia in two patients (at 10 μg/kg/min), and worsening hypotension with new-onset atrial fibrillation in one patient (at 5 μg/kg/min).

**Figure 1:**
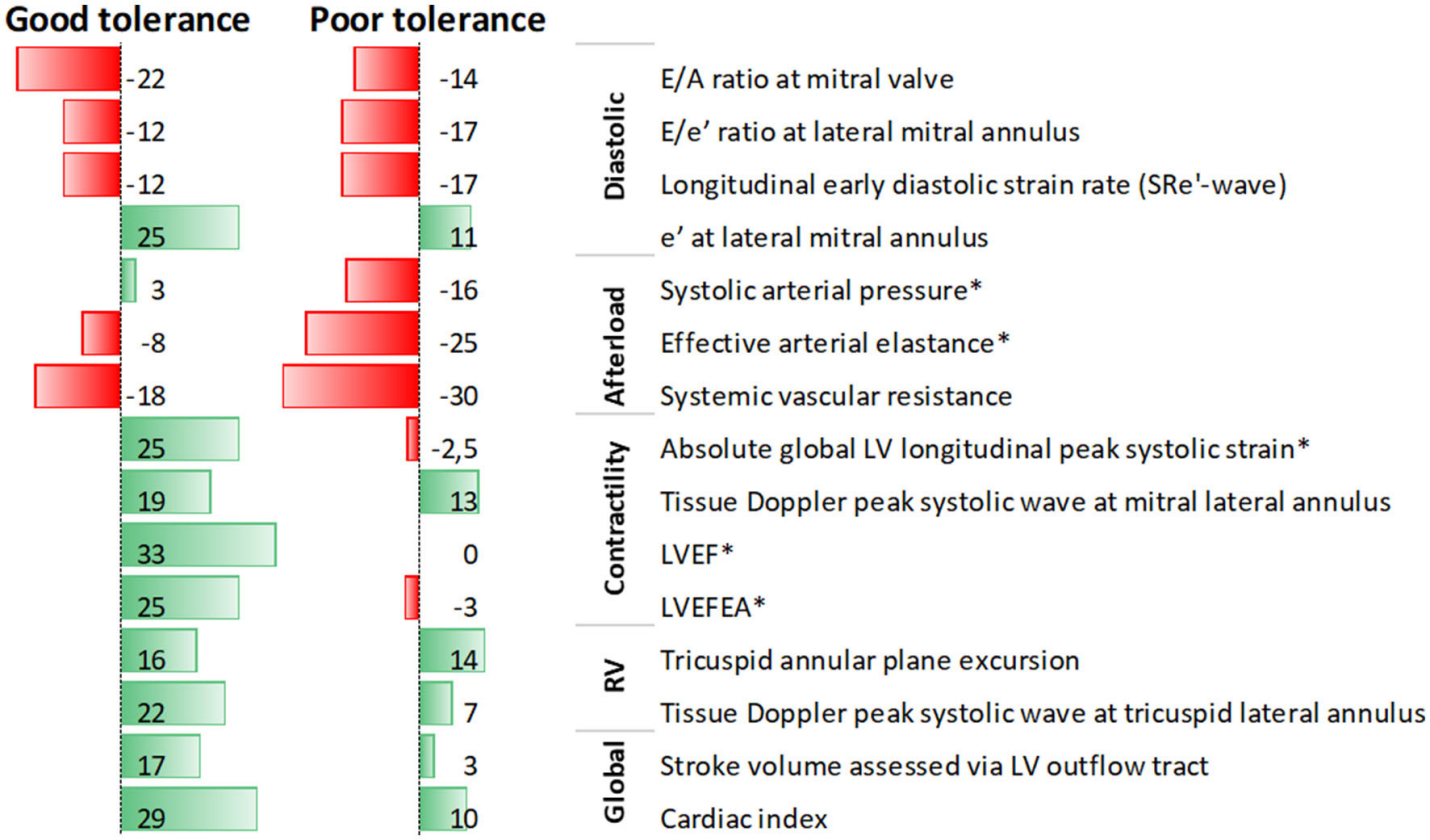
Data bars of median values of percent change in echocardiographic parameters after low-dose dobutamine infusion in septic shock patients with septic myocardial dysfunction, according to tolerance to dobutamine; *denotes significant difference between patients with good and poor tolerance.

After titration, dobutamine was maintained during septic shock treatment by the attending intensivist for a median of 2.5 [1–3] days, and continuation after titration was more frequent in patients with a good tolerance to low-dose dobutamine (5 μg/kg/min) than in their counterparts with poor tolerance [14/17 (82%) vs. 7/15 (47%), *p* = 0.03]. Supplementary Tables S1, S2 compare patients with good or poor tolerance to low-dose dobutamine in terms of baseline characteristics or percent change of circulatory parameters after dobutamine infusion, respectively. Patients with a good tolerance to low-dose dobutamine had a greater improvement in contractility indices whereas those with poor tolerance had a more severe deterioration of afterload indices (Supplementary Table S1, Figure 1). At baseline, clinical characteristics, hemodynamics, and echocardiographic parameters did not differ between patients with good or poor tolerance to low-dose dobutamine, except for a lower arterial blood pH in patients with poor tolerance (Supplementary Table S2).

### Effect of acidosis

Patients with lesser acidosis (pH ≥ 7.28, i.e., the median value of the cohort) had more improvement in global LV longitudinal peak systolic strain, ventricular–arterial coupling, oxygen transport, and oxygen consumption with dobutamine whereas those with greater acidosis (pH <7.28) had a more severe deterioration of systolic, diastolic and mean arterial pressure with dobutamine (Supplementary Table S3 and Supplementary Figure S1).

### Outcome

At day-14, patients with a good tolerance to low-dose dobutamine had more vasopressor-free days (11 [0–13] vs. 0 [0–8] days, *p* = 0.01) and a lower mortality [4 (24%) vs. 9 (60%), *p* = 0.04] than their counterparts. However, ICU mortality was not significantly different between groups [6 (35%) vs. 9 (60%), *p* = 0.16].

## Discussion

In this study of patients having septic myocardial dysfunction and severe septic shock, we have evidenced the following findings: i) dobutamine improved echocardiographic parameters of diastolic and biventricular systolic function, while it decreased afterload; ii) nearly half and two thirds of patients had a poor tolerance to low-dose and to any dose of dobutamine, respectively; the inotropic effect was prominent in patients with good tolerance to low-dose dobutamine, while poor tolerance was enhanced by acidosis, and associated with deteriorated vasoplegic response and a worse short-term prognosis.

### Systolic function

Dobutamine is a synthetic catecholamine that was developed as an inotrope for use in congestive heart failure. It consists of two composite enantiomers, which explains its mixed action on α1, α2, β1, and β2 receptors (30). Variability in LVEF during septic shock may mainly reflect the influence of loading conditions (2). Indeed, LVEF and other systolic indices reflect the ventriculo-arterial coupling between LV contractility and LV afterload. In our study, while dobutamine decreased systemic afterload, the improvement in LV contractility was not ascribable to the decrease in afterload. Indeed, among contractility parameters, global LV longitudinal peak systolic strain, longitudinal systolic strain rate, tissue Doppler peak systolic wave at the lateral mitral valve annulus (which are less dependent from loading conditions) increased after dobutamine infusion. Moreover, dobutamine induced a significant increase in the afterload-adjusted LVEF.

### Diastolic function

Myocardial cyclic adenosine monophosphate (cAMP) is increased by dobutamine's β-adrenoreceptors stimulation. Intracellular calcium increases secondary to elevated cAMP concentrations and could exacerbate diastolic dysfunction (31). The tachycardic response to dobutamine could also favor diastolic dysfunction. Indeed, sepsis impairs frequency-dependent acceleration of relaxation, which normally maintains appropriate ventricular filling at high heart rates through the acceleration of sarcoplasmic/endoplasmic reticulum calcium ATPase (SERCA) activity (32). However, cAMP also mediate the effect of β-adrenergic receptor stimulation to cause myocardial relaxation (i.e., positive lusitropic effect) (33). Moreover, studies in muscle strips (34), isolated hearts (35), and intact animal (36), have demonstrated that β-adrenergic receptor stimulation accelerates myocardial relaxation. Contrary to our hypothesis, we found that the net effect of dobutamine on diastolic function was an improved relaxation. These results are in accordance with those observed in patients with severe chronic heart failure (33).

### Mottling and tissue hypoxia

Previous findings suggested beneficial effects of dobutamine on microcirculation (37). We only found a trend toward a reduction in mottling score, but our results may be affected by the limited sample size and the low values of mottling score at baseline in our cohort. Some patients with significant microcirculatory alterations cannot be identified by visual assessment (38). Moreover, both favorable and neutral effects of dobutamine on microcirculatory parameters have been reported (7, 37). Our results are in accordance with previous study with indirect calorimetry showing an increase in VO2 and VCO2 after dobutamine infusion, with stable respiratory quotient (39).

### Dobutamine tolerance, acidosis, and outcome

In our study, dobutamine was discontinued because of worsening hypotension or tachycardia in nearly two-thirds of patients at any dose (5 to 15 μg/kg/min) and in nearly half of patients at low-dose (5 μg/kg/min). This result is in accordance with a previous monocenter study with dobutamine incremental doses (6). Patients with poor tolerance to low-dose dobutamine had a mitigated inotropic response and an enhanced vasoplegic response to dobutamine. Baseline characteristics were not different between patients with good or poor tolerance to low-dose dobutamine, except for more severe acidosis in the latter group. Acidosis has been shown to impair cardiac function. The drop in pH reduces the number of myocardial beta-adrenoreceptors (40), and decreases the affinity of catecholamine for the beta-adrenoreceptor (41). Acidosis also induce vascular smooth muscle relaxation via the opening of ATP-sensitive potassium channels and vasodilatation secondary to overproduction of nitric oxide by inductible NO synthase (42, 43). Acidosis may therefore impair the inotropic response and worsen the vasoplegic response to dobutamine, altering its tolerance. The effect of acidosis correction on dobutamine response needs to be assessed.

In our study, a poor tolerance of dobutamine was associated with a worse outcome. A favorable response to dobutamine infusion (in terms of oxygen delivery to the tissues or whole body oxygen consumption) has been associated with a better outcome (44). A recent meta-analysis suggested that the combination of norepinephrine and dobutamine is associated with a reduction in mortality at day-28 in patients with septic shock and low cardiac output (45). Such results should be considered carefully since the heterogeneity of the studies included remains high. Randomized controlled trial are ongoing to assess dobutamine in septic shock patients with myocardial dysfunction and low cardiac output (46).

### Strengths and limitations

The strengths of our study include its prospective and multicentre design, the careful selection of patients with septic myocardial dysfunction, the comprehensive hemodynamic phenotyping of cardiac function (with evaluation of preload, contractility and afterload with advanced tools including strain imaging).

Our study has several limitations. The sample size was rather small, we explored patients within a short period and most patients were included in one center. We cannot exclude that some patients had chronic heart failure and/or baseline diastolic dysfunction. We cannot exclude that the statistical power and/or intra or interobserver variability and/or the limited period of observation were insufficient to detect subtle differences in some variables. We cannot exclude that tolerance of dobutamine would have been better with a lower dose of dobutamine such as 2.5 μg/kg/min, especially in patients with severe acidosis. Although fluid responsiveness was assessed before starting dobutamine, we cannot exclude that some degree of hypovolemia may have worsened dobutamine tolerance (47). We could not assess PCO_2_ gap as a marker of cardiac output adequacy as many patients had the central venous catheter in femoral position. In our study, LV end systolic volume was not directly measured, but derived from ejection fraction and stroke volume, which may induce errors in LV efficiency assessment. Our finding of LV efficiency impairment with dobutamine warrants confirmation, as a previous small sample size study showed no effect of dobutamine on LV efficiency (48).

## Conclusion

Dobutamine improved echocardiographic parameters of diastolic and biventricular systolic function, but further decreased LV afterload in human sepsis with myocardial dysfunction. Oxygen transport, oxygen consumption and carbon dioxide production both increased, with a stable respiratory quotient. Dobutamine titration was poorly tolerated in a majority of patients, with worsening hypotension. Poor tolerance to low-dose dobutamine was enhanced by acidosis, mediated by worsened vasoplegic response and associated with lower vasopressor-free days and survival at day-14. These results may suggest that dobutamine should be infused at low dose, especially in patients with severe acidosis.

## Data availability statement

The raw data supporting the conclusions of this article will be made available by the authors, upon reasonable request.

## Ethics statement

The studies involving human participants were reviewed and approved by Institutional Review Board (CPP Ile de France-V). Written informed consent for participation was not required for this study in accordance with the national legislation and the institutional requirements.

## Author contributions

KR had full access to all of the data in the study and takes responsibility for the integrity of the data and the accuracy of the data analysis. KR and AMD contributed to initial study design, analysis, interpretation of data, drafting of the submitted article, critical revisions for intellectual content, and providing final approval of the version to be published. VL, LL, AB, GC, NP, FBo, and FBa contributed to study design and analysis, interpretation of data, drafting of the submitted article, critical revisions for intellectual content, and providing final approval of the version to be published. All authors contributed to the article and approved the submitted version.

## Funding

This trial was supported by French intensive care society (SRLF), for financial support of a fellow.

## Conflict of interest

Author VL receives advisory board fees from Amomed and grant from Leopharma, unrelated to the present study. The remaining authors declare that the research was conducted in the absence of any commercial or financial relationships that could be construed as a potential conflict of interest.

## Publisher's note

All claims expressed in this article are solely those of the authors and do not necessarily represent those of their affiliated organizations, or those of the publisher, the editors and the reviewers. Any product that may be evaluated in this article, or claim that may be made by its manufacturer, is not guaranteed or endorsed by the publisher.

## Abbreviations

LVEF: left ventricle ejection fraction
SAPS II: Simplified Acute Physiologic Score
SOFA: Sequential Organ Failure Assessment.
