# Supplementary material for: Hemodynamic effects and tolerance of dobutamine for myocardial dysfunction during septic shock: An observational multicenter prospective echocardiographic study
Source: Front Cardiovasc Med. 2022 Sep 9;9:951016. doi: 10.3389/fcvm.2022.951016 (PMC9500364; doi:10.3389/fcvm.2022.951016)
Supplement: Supplementary file 1 [file Data_Sheet_1.docx]

Table S1. Percent change in hemodynamic parameters after low-dose dobutamine infusion (5 γ /kg/min) according to its clinical tolerance in patients with septic shock and myocardial dysfunction

| p | **Good tolerance**  **(n=17)** | **Poor tolerance**  **(n=15)** | **P value** |
| --- | --- | --- | --- |
| **Macrocirculation** |  |  |  |
| Percent change in dose of norepinephrine µg.kg^-1^.min^-1^ | 0 [0- 0] | 0 [0 -14] | 0.004 |
| Percent change in dose of norepinephrine mg/h | 0 [0- 0] | 0 [0 -14] | 0.004 |
| Percent change in mean arterial blood pressure | 1 [-7 to 12] | -19 [-25 to -15] | <0.001 |
| Percent change in heart rate | 7 [0-13] | 5 [1-7] | 0.62 |
| Percent change in diastolic arterial pressure | -2 [-8 to 4] | -20 [-24 to -15] | <0.001 |
| Percent change in diastolic shock index | 10 [1- 22] | 34 [25-48] | <0.001 |
| **Echocardiography** |  |  |  |
| Percent change in respiratory variation of inferior vena cava | -8 [-14 to -2] | -12 [-29 to -2] | 0.35 |
| ***Diastolic function*** |  |  |  |
| Percent change in E/A ratio at mitral valve | -22 [-43 to 10] | -14 [-30 to 13] | 0.55 |
| Percent change in E-wave deceleration time | 7.6 [-18; 31] | -10 [-23; 28] | 0.68 |
| Percent change in E/e’ ratio at lateral mitral annulus | -12 [-32 to 6] | -17 [-23 to 13] | 0.63 |
| Percent change e’ at lateral mitral annulus | 25 [0 to 59] | 11 [-2 to 30] | 0.23 |
| Percent change longitudinal SRe′- wave | 37 [10-94] | 8 [-8 to 30] | 0.07 |
|  |  |  |  |
| ***Contractili*ty** |  |  |  |
| Percent change in pulse pressure | 20 [-1 to 29] | -20 [-31 to 4] | 0.002 |
| Percent change in absolute global LV longitudinal peak systolic strain | 25 [16 to 41] | -2.5 [-10 to 16] | 0.02 |
| Percent change in longitudinal SRs′-wave | 47 [27-81] | 14 [7-60] | 0.07 |
| Percent change in tissue Doppler peak systolic wave at mitral lateral annulus | 19 [-3 to 44] | 13 [-8 to 50] | 0.90 |
| Percent change in LVEF | 33 [24- 50] | 0 [0- 35] | 0.02 |
| Percent change in afterload-adjusted LVEF | 25 [16- 41] | -3 [-10 to 16] | 0.001 |
| Percent change in LV end-systolic elastance | 16 [-18 to 37] | -12 [-41 to -7] | 0.07 |
| ***Afterload*** |  |  |  |
| Percent change in systolic arterial pressure | 3 [-9 to 15] | -16 [-24 to -3] | 0.03 |
| Percent change in effective arterial elastance | -8 [-19 to -2] | -25 [-37 to -9] | 0.02 |
| Percent change in systemic vascular resistance | -18 [-37 to -9] | -30 [-39 to -20] | 0.17 |
| ***RV function*** |  |  |  |
| Percent change in tricuspid annular plane excursion | 16 [-8 to 27] | 14 [-16 to 35] | 0.09 |
| Percent change in tissue Doppler peak systolic wave at tricuspid lateral annulus | 22 [9- 61] | 7 [1- 28] | 0.06 |
| Percent change in RV/LV area ratio | 0 [0- 0] | 0 [-9 to 0] | 0.23 |
| ***Global function*** |  |  |  |
| Percent change in stroke volume assessed via LV outflow tract | 14 [3- 35] | 2 [0- 21] | 0.08 |
| Percent change in cardiac index | 29 [6-50] | 10 [5- 29] | 0.21 |
| Percent change in ventricular–arterial coupling | -12 [-28 to -1] | 8 [-24 to 49] | 0.20 |
| Percent change in stroke work (mmHg mL) | 21 [13-42] | - 12 [-24 to -4] | <0.001 |
| Percent change in potential energy (mmHg mL) | 120 [67-172] | - 4 [-20 to 65] | 0.008 |
| Percent change in LV efficiency (%) | -11 [-19 to -6] | -2 [-10-0] | 0.02 |
| **Arterial blood gas** |  |  |  |
| Percent change in PaO_2_/FiO_2_ | -6 [-12- 5] | -2 [-15 to 10] | 0.88 |
| Percent change in SaO_2_ | 0 [-1 to 1] | 0 [-3 to 1] | 0.87 |
| Percent change in arterial blood lactates | -3 [-8 to 3] | -3 [-11 to 6] | 0.83 |
| **Oxygen metabolism** |  |  |  |
| Percent change in oxygen transport | 29 [5-52] | 8 [3-33] | 0.21 |
| Percent change in oxygen consumption | 7 [3- 11] | -4 [-1 to -4] | 0.01 |
| Percent change in energy expenditure | 7 [3; 9] | -1 [-4 to -1] | 0.03 |
| Percent change in carbon dioxide production | 4 [-1 to 7] | 3 [-8 to 3] | 0.99 |

LV, left ventricle; RV, right ventricle; LVEF, left ventricle ejection fraction ; PaO_2_, partial pressure of oxygen tension in arterial blood ; FiO_2_, fraction of inspired oxygen ;TaO_2_, oxygen transport.

Table S2. Baseline clinical, hemodynamic, echocardiographic, calorimetric and arterial blood gases data in patients with shock and septic myocardial dysfunction according to clinical tolerance at low-dose of dobutamine (5 γ /kg/min).

|  | **Good tolerance**  **(n=17)** | **Poor tolerance #**  **(n=15)** | **P value** |
| --- | --- | --- | --- |
| Age, years | 64 [44-73] | 69 [66-77] | 0.17 |
| Male gender | 9 (53%) | 10 (67%) | 0.49 |
| Body mass index, kg/m^2^ | 24 [19-27] | 23 [18-25] | 0.56 |
| SAPS II at ICU admission | 57 [45-70] | 73 [55-85] | 0.052 |
| SOFA on the day of dobutamine initiation | 11 [10-13] | 12 [11- 14] | 0.11 |
| Delay between ICU admission and dobutamine initiation, days | 1.0 [0-1.5] | 0 [0- 1] | 0.06 |
| Fluid administration before dobutamine initiation, ml | 2750 [1625-6188] | 2750 [1500-3313] | 0.57 |
| **Macrocirculation** |  |  |  |
| Dose of norepinephrine, µg.kg^-1^.min^-1^ | 0.9 [0.2-2.1] | 1.6 [1.0- 2.4] | 0.17 |
| Dose of norepinephrine, mg/h | 3.9 [1.1-8.5] | 6.2 [3.2- 11.9] | 0.29 |
| Mean arterial blood pressure, mmHg | 75 [73-80] | 72 [67- 75] | 0.13 |
| Diastolic arterial pressure, mmHg | 59 [56-65] | 55 [50- 63] | 0.20 |
| Heart rate, bpm | 100 [79-112] | 105 [85- 121] | 0.19 |
| Diastolic shock index, bpm. mmHg^-1^ | 1.6 [1.4-1.9] | 1.9 [1.5- 2.3] | 0.07 |
| **Echocardiography** |  |  |  |
| Respiratory variation of inferior vena cava, % | 6 [0-12] | 0 [0-10] | 0.32 |
| ***Diastolic function*** |  |  |  |
| E/A ratio at mitral valve | 0.95 [0.61-1.13] | 0.91 [0.73-1.22] | 0.79 |
| e’ at lateral mitral annulus, cm. s^-1^ | 5.0 [4.0-9.0] | 7.0 [4.3-9.7] | 0.41 |
| E/e’ ratio at lateral mitral annulus | 8.1 [5.9-13.1] | 8.3 [6.9-14.8] | 0.95 |
| Longitudinal SRe′- wave | 0.64 [0.56-1.1] | 0.71 [0.56-0.76] | 0.99 |
| ***Contractili*ty** |  |  |  |
| Global LV longitudinal peak systolic strain, % | -9.0 [-11.8 to -5.0] | -8.4 [-10.1 to -7.3] | 0.99 |
| Longitudinal SRs′-wave, | -0.72 [-0.96 to -0.60] | -0.71 [-0.85 to -0.54] | 0.94 |
| s’at mitral lateral annulus, cm.s^-1^ | 8.0 [7.0-11.2] | 8 [5.0-10.6] | 0.58 |
| LVEF, % | 30 [25- 40] | 30 [20-40] | 0,79 |
| Afterload-adjusted LVEF, % | 43 [33-60] | 39 [33- 51] | 0.68 |
| LV end-systolic elastance, mmHg.mL^-1^ | 1.8 [1.0-2.2] | 1.4 [1.0-2.1] | 0.38 |
| ***Afterload*** |  |  |  |
| Systolic arterial pressure, mmHg | 108 [97-118] | 113 [104-131] | 0.39 |
| Effective arterial elastance~~,~~ mmHg.mL^-1^ | 2.8 [2.1-3.1] | 2.6 [2.1- 3.3] | 0.91 |
| Systemic vascular resistance, mmHg.L^-1^.min | 1732 [1427-2115] | 1448 [1036- 2137] | 0.17 |
| ***RV function*** |  |  |  |
| Tricuspid annular plane excursion, mm | 15 [12-17] | 15 [14-17] | 0.91 |
| Tissue Doppler peak systolic wave at tricuspid lateral annulus, cm/s | 10.1 [7.7-11.6] | 11.3 [7.8-14.1] | 0.53 |
| RV dilatation (RV/LV area ratio) | 0.6 [0.5-0.7] | 0.6 [0.5-0.6] | 0.77 |
| ***Global function*** |  |  |  |
| Stroke volume assessed via LV outflow tract, mL | 37 [31-44] | 37 [28- 48] | 0.99 |
| Cardiac output, L.min^-1^ | 3.6 [2.7-4.4] | 4.0 [2.7-5.8] | 0.55 |
| Cardiac index, L.min^-1^.m^-2^ | 2.0 [1.5-2.7] | 2.2 [1.8-3.3] | 0.39 |
| Ventricular–arterial coupling | 1.6 [1.2-2.1] | 2.1 [1.6-2.4] | 0.17 |
| Stroke work (mmHg mL) | 3502 [3042-4353] | 3483 [2642-5078] | 0.97 |
| Potential energy (mmHg mL) | 1090 [496-1483] | 846 [400-1518] | 0.97 |
| LV efficiency (%) | 82 [75-86] | 82 [75-89] | 0.80 |
| **Mottling** | 3 (21%) | 6 (40%) | 0.14 |
| **Arterial blood gas** |  |  |  |
| pH | 7.32 [7.27-7.39] | 7.21 [7.18-7.26] | 0.02 |
| Bicarbonates, mmol/L | 19 [16-22] | 16 [14-21] | 0.27 |
| PaCO_2_, mmHg | 37 [30-39] | 40 [29-46] | 0.20 |
| PaO_2_/FiO_2_ ratio, mmHg | 203 [125-314] | 214 [120-350] | 0.93 |
| SaO_2_, % | 96 [94-98] | 97 [94-98] | 0.89 |
| Lactates, mmol/L | 2.3 [1.4-3.2] | 3.3 [1.9-7.0] | 0.15 |
| **Oxygen metabolism** |  |  |  |
| TaO_2_, mL.min^-1^.m^-2^ | 319 [208-387] | 309 [278-414] | 0.68 |
| VO_2_, ml/min | 252 [194-275] | 163 [112-340] | 0.38 |
| VCO_2_, ml/min | 177 [138-188] | 143 [98-187] | 0.71 |
| Respiratory quotient | 0.70 [0.68-0.73] | 0.71 [0.55-0.88] | 0.99 |
| Energy expenditure, kcal/day | 1670 [1295-1806] | 1478 [759-1572] | 0.28 |
| **Outcome** |  |  |  |
| Days alive and free of norepinephrine at day 14 | 11 [0-13] | 0 [0-8] | 0.01 |
| Death in intensive care unit | 6 (35%) | 9 (60%) | 0.16 |

Data are number (percentage) or median [1^st^ quartile – 3^rd^ quartile]; #poor tolerance was defined as one of the following: i) worsening hypotension (mean arterial pressure < 65 mmHg with decrease of 10 mmHg or more as compared to baseline or the need to increase norepinephrine infusion to maintain a mean arterial pressure of 65 mmHg or more); ii) worsening tachycardia (increase of 10 beats per minute and sinus tachycardia > 130 mmHg or new-onset atrial fibrillation); SAPS, simplified acute physiologic score; SOFA, Sequential Organ Failure Assessment; LV, left ventricle ; RV, right ventricle; LVEF, left ventricle ejection fraction; TaO_2_, Oxygen transport; VO_2_ oxygen consumption determined by indirect calorimetry, VCO_2_: carbon dioxide production determined by indirect calorimetry; E, blood Doppler early diastolic wave; A, blood Doppler late diastolic wave; e’, tissue Doppler early diastolic wave; s’, tissue Doppler peak systolic wave;; see text for definitions.

Table S3 (online supplement). Percent change in hemodynamic parameters after low-dose dobutamine infusion (5 γ /kg/min) according to the presence or not of severe acidosis (pH<7.28)

| p | **pH≥7.28**  **(n=16)** | **pH<7.28**  **(n=16)** | **P value** |
| --- | --- | --- | --- |
| **Macrocirculation** |  |  |  |
| Percent change in dose of norepinephrine µg.kg^-1^.min^-1^ | 0 [0-0] | 0 [0-7] | 0.33 |
| Percent change in dose of norepinephrine mg/h | 0 [0-0] | 0 [0-10] | 0.33 |
| Percent change in mean arterial blood pressure | 1 [-10 to 10] | -17 [-25 to -8] | 0.002 |
| Percent change in heart rate | 7 [1-17] | 5 [-1 to 7] | 0.29 |
| Percent change in diastolic arterial pressure | -3 [-14 to 1] | -18 [-24 to -11] | 0.002 |
| Percent change in diastolic shock index | 12 [0-27] | 25 [19-39] | 0.04 |
| **Echocardiography** |  |  |  |
| Percent change in respiratory variation of inferior vena cava | 12 [0-24] | 2 [0-18] | 0.25 |
| ***Diastolic function*** |  |  |  |
| Percent change in E/A ratio at mitral valve | -7 [-24 to 7] | 3 [-13 to 17] | 0.25 |
| Percent change in E/e’ ratio at lateral mitral annulus | -15 [-27 to -3] | -16 [-25 to 10] | 0.72 |
| Percent change in e’ at lateral mitral annulus | 28 [5 to 48] | 1 [-10 to 31] | 0.11 |
| Percent change in longitudinal peak early diastolic strain-rate | 37 [7-131] | 16 [-2 to 32] | 0.35 |
| ***Contractili*ty** |  |  |  |
| Percent change in pulse pressure | 7 [-19 to 28] | -15 [-25 to 7] | 0.14 |
| Percent change in absolute global LV longitudinal peak systolic strain | 72 [28-134] | 9 [-6 to 24] | 0.003 |
| Percent change in longitudinal peak systolic strain-rate | 47 [22-110] | 24 [13-61] | 0.35 |
| Percent change in tissue Doppler peak systolic wave at mitral lateral annulus | 22 [-5 to 54] | 13 [-2 to 38] | 0.78 |
| Percent change in LVEF | 33 [21-45] | 25 [0-50] | 0.53 |
| Percent change in afterload-adjusted LVEF | 20 [10-29] | 13 [-7 to 33] | 0.30 |
| Percent change in LV end-systolic elastance | 1 [-20 to 42] | -9 [-41 to 13] | 0.17 |
| ***Afterload*** |  |  |  |
| Percent change in systolic arterial pressure | 3 [-9 to 15] | -15 [-24 to -3] | 0.04 |
| Percent change in effective arterial elastance | -8 [-34 to -4] | -10 [-33 to -2] | 0.89 |
| Percent change in systemic vascular resistance | -22 [-37 to -12] | -25 [-36 to -12] | 0.60 |
| ***RV function*** |  |  |  |
| Percent change in tricuspid annular plane excursion | 17 [0-32] | 6 [-21 to 27] | 0.23 |
| Percent change in tissue Doppler peak systolic wave at tricuspid lateral annulus | 20 [8-46] | 9 [0 -42] | 0.35 |
| Percent change in RV/LV area ratio | 0 [0-0] | 0 [0-0] | 0.12 |
| ***Global function*** |  |  |  |
| Percent change in stroke volume assessed via LV outflow tract | 18 [2-27] | 5 [0-13] | 0.07 |
| Percent change in cardiac index | 28 [9-45] | 10 [5-40] | 0.13 |
| Percent change in ventricular–arterial coupling | -16 (±24) | 12 (±29) | 0.02 |
| Percent change in stroke work(mmHg mL) | 19 [-5 to 43] | -8 [-25 to 21] | 0.02 |
| Percent change in potential energy (mmHg mL) | 84 [51-127] | 32 [-18 to 135] | 0.16 |
| Percent change in LV efficiency (%) | -9 [-19 to -4] | -5 [-14 to 0] | 0.38 |
| **Arterial blood gas** |  |  |  |
| Percent change in PaO_2_/FiO_2_ | 4 [-6 to 16] | -13 [-16 to -4] | 0.01 |
| Percent change in SaO_2_ | 0 [0-1] | -1 [-4 to 1] | 0.09 |
| Percent change in arterial blood lactates | -2 [-8 to 0] | -3 [-10 to 9] | 0.92 |
| **Oxygen metabolism** |  |  |  |
| Percent change in oxygen transport | 30 [9-46] | 6 [2- 41] | 0.07 |
| Percent change in oxygen consumption | 7 [4-12] | 0 [-2 to 6] | 0.03 |
| Percent change in energy expenditure | 7 [3-11] | 1 [-2 to 5] | 0.03 |
| Percent change in carbon dioxide production | 4 [3-7] | -2 [-7 to 9] | 0.25 |

LV, left ventricle; RV, right ventricle; LVEF, left ventricle ejection fraction ; PaO_2_, partial pressure of oxygen tension in arterial blood ; FiO_2_, fraction of inspired oxygen ;TaO_2_, oxygen transport.

Table S4 (online supplement). Reproducibility of echocardiography parameters

| **Parameter** | British Standards Institution coefficient coefficient |
| --- | --- |
| e’ at lateral mitral annulus, cm.s^-1^ | 1.3 |
| s’ at mitral lateral annulus, cm.s^-1^ | 1.6 |
| Global LV longitudinal peak systolic strain, % | 1.3 |
| Velocity time integral, cm | 1.5 |
| LV end systolic elastance, mmHg.mL-1 | 0.26 |
