# Supplementary figures and images for: Hemodynamic effects and tolerance of dobutamine for myocardial dysfunction during septic shock: An observational multicenter prospective echocardiographic study
Source: Front Cardiovasc Med. 2022 Sep 9;9:951016. doi: 10.3389/fcvm.2022.951016 (PMC9500364; doi:10.3389/fcvm.2022.951016)

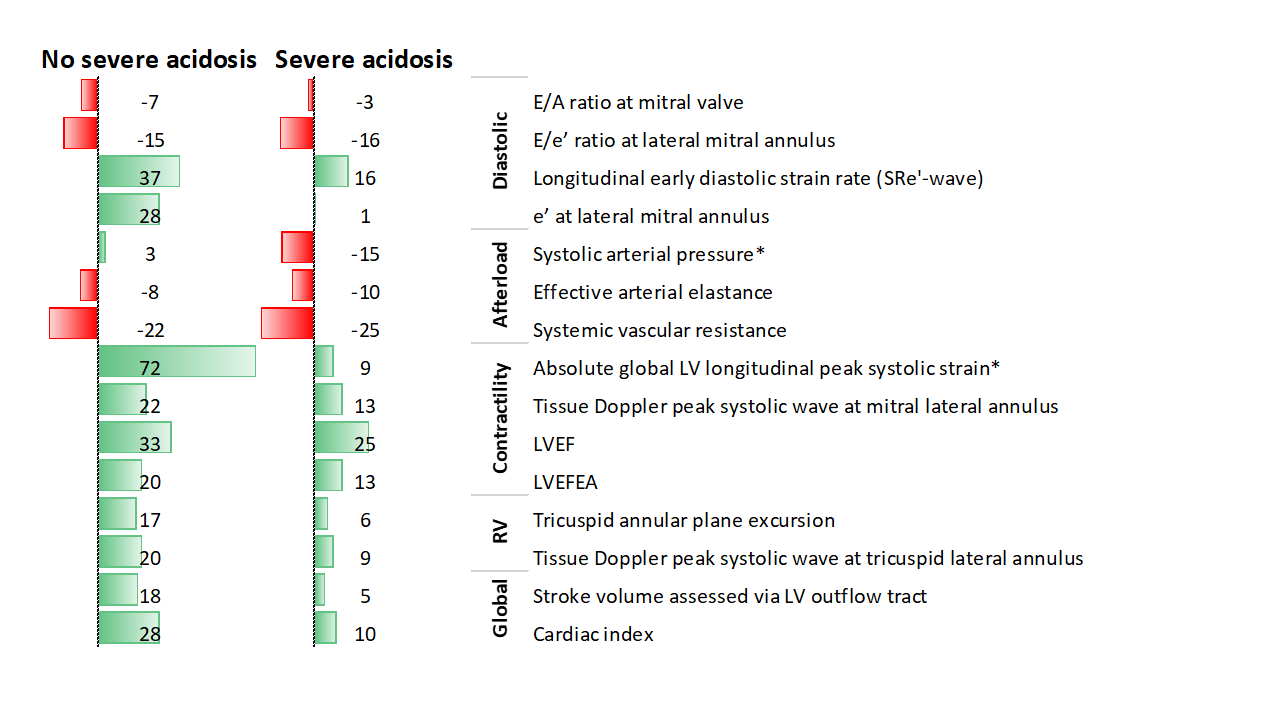

Supplement: Supplementary file 2 [file Image_1.tif]
